# X-rays Activate Telomeric Homologous Recombination Mediated Repair in Primary Cells

**DOI:** 10.3390/cells8070708

**Published:** 2019-07-12

**Authors:** Marco De Vitis, Francesco Berardinelli, Elisa Coluzzi, Jessica Marinaccio, Roderick J. O’Sullivan, Antonella Sgura

**Affiliations:** 1 Department of Science, University of Rome “ROMA TRE”, 00146 Rome, Italy; 2 Department of Pharmacology & Chemical Biology, UPMC Hillman Cancer Center, Pittsburgh, PA 15232, USA

**Keywords:** telomere, ALT, DNA repair, homologous recombination, oxidative stress, ionizing radiations

## Abstract

Cancer cells need to acquire telomere maintenance mechanisms in order to counteract progressive telomere shortening due to multiple rounds of replication. Most human tumors maintain their telomeres expressing telomerase whereas the remaining 15%–20% utilize the alternative lengthening of telomeres (ALT) pathway. Previous studies have demonstrated that ionizing radiations (IR) are able to modulate telomere lengths and to transiently induce some of the ALT-pathway hallmarks in normal primary fibroblasts. In the present study, we investigated the telomere length modulation kinetics, telomeric DNA damage induction, and the principal hallmarks of ALT over a period of 13 days in X-ray-exposed primary cells. Our results show that X-ray-treated cells primarily display telomere shortening and telomeric damage caused by persistent IR-induced oxidative stress. After initial telomere erosion, we observed a telomere elongation that was associated to the transient activation of a homologous recombination (HR) based mechanism, sharing several features with the ALT pathway observed in cancer cells. Data indicate that telomeric damage activates telomeric HR-mediated repair in primary cells. The characterization of HR-mediated telomere repair in normal cells may contribute to the understanding of the ALT pathway and to the identification of novel strategies in the treatment of ALT-positive cancers.

## 1. Introduction

Telomeres are specialized nucleoprotein complexes that protect the ends of linear eukaryotic chromosomes from degradation, erosion, promiscuous recombinogenic events and end-to-end ligations that result in fusion with other chromosomes [1,2,3]. Human telomeres contain 7–20 kilobase pairs (kbp) of tandem arrays of TTAGGG repeats [4,5,6] bound by two specific TAAGGG DNA-binding proteins, namely repeat binding factor 1 and 2 (TRF1 and TRF2, respectively) [7]. These are essential to maintain functional telomeres, and together with RAP1, TIN2, TPP1, and POT1 constitute the Shelterin complex. TRF2, in particular, has been implicated in the formation of a telomeric higher order structure, the t-loop [8,9].

The role of the Shelterin complex and the t-loop is to conceal chromosome ends from DNA repair machinery that might recognize them as DNA double strand breaks (DSBs). However, the suppression of DNA repair at telomeres might also become detrimental in the case of DSBs inside telomeric regions. Evidence from the literature suggest that the heterochromatic nature of telomeric chromatin [10] and/or inhibition of non-homologous end-joining (NHEJ) by TRF2 [11,12,13] reduce DNA DSBs repair efficiency in telomeres [14,15]. Moreover, as recently shown, telomere repair mechanisms may also be affected by the replicative state of the cell [16].

Due to their guanine-rich DNA sequences, telomeres are highly prone to oxidative attacks induced by a number of DNA damaging agents [15,17], and emerging data indicate that homologous recombination (HR)-mediated processes may be primarily responsible for their repair. Interestingly, a subset of cancer cells activates the telomerase-independent alternative lengthening of telomere (ALT) pathway. ALT is a HR-based pathway for exchange and/or synthesis of telomeric DNA [18,19]. This involves the invasion of the resected 3′ DNAs at DNA lesions or collapsed replication forks within duplex telomeric DNA on sister chromatids or other chromosomes. This primes homology directed repair mechanisms that repair damaged telomeric DNA and initiate DNA synthesis, yielding to a net gain in telomere length.

Well-known hallmarks of ALT activation include heterogeneous telomere length [20,21], a high level of telomere-sister chromatid exchanges (T-SCEs) [18], extrachromosomal telomeric repeats DNA (ECTRs) [22,23], and a specialized telomeric nuclear structure called ALT-associated PML (promyelocytic leukemia protein) bodies (APBs) [24]. It has been demonstrated that different factors could be responsible for ALT activation. Somatic mutations in the genes encoding for the α-thalassemia/mental retardation syndrome X-linked proteins (ATRX) and the death domain-associated protein (DAXX) chromatin remodeling complex (that modulates chromatin changes including telomeric chromatin during the S-phase) [25], together with H3.3 histone mutations, appear to be highly related to ALT-positive tumors [26,27,28,29]. Moreover, it was demonstrated that depletion of the gene encoding the anti-silencing factor 1 (ASF1), a histone chaperone that assists in the transfer of H3.1–H4 or H3.3–H4 histone dimers for nucleosome assembly [30], can lead to the manifestation of all phenotypes consistent with the activation of telomere maintenance by the ALT pathway, including APBs, ECTRs, T-SCEs and telomere length heterogeneity [31]. The defective chromatin assembly triggered by ASF1 depletion could impact the transmission of epigenetic histone modification [32], activating other genes related to ALT [31]. These data provide a biological role for a specific chromatin organization in ALT cells. In fact, it has been speculated that alterations in such chromatin factors may create a chromatin environment that is more permissive to HR activities that are normally repressed. Furthermore, several findings have indicated that ALT telomeres are prone to replication stress and that DSBs (induced or caused by replication forks collapse) can give rise to a break-induced telomere synthesis, leading to inter- or intra-telomeric recombination via HR, and so to telomere elongation [33,34]. Indeed, there are several studies that highlight a link between ALT pathway activation and telomere damage. For example, it has been shown that mortal cells, telomerase- or ALT-positive cells could respectively show [16,31,35] or enhance [33,36] an ALT phenotype after telomeric damage induction, demonstrating a simultaneous and reduced localization of signaling proteins of the DNA damage response (DDR) at telomeres [16]. Finally, studies indicated that IR transiently induced some of the ALT hallmarks in somatic normal cells [37] to cope with DNA damage [16].

In this work, we exposed human foreskin fetal fibroblasts (HFFF2) to X-rays and evaluated telomere length modulation, DNA damage, and the frequency of ALT hallmarks over a period of 10 days. 

Our results showed that X-ray-treated cells displayed telomere shortening and telomeric damage caused by ionizing radiation-induced persistent oxidative stress (OS). After initial telomere erosion we observed a telomere elongation that was associated to the transient activation of a HR-based mechanism, sharing several features with the ALT pathway observed in cancer cells. Data provided strongly indicate that IR-dependent telomeric damage activates telomeric HR-mediated repair in normal primary cells. The implications of these results are discussed.

## 2. Materials and Methods

### 2.1. Cell Culture

HFFF2 cell line (ECACC, Salisbury, UK) was cultured in Dulbecco’s Modified Eagle Medium supplemented with 10% fetal bovine serum (FBS), 100 units/mL penicillin, 100 µg/mL streptomycin, and 2 mM l-glutamine (Euroclone, Milan, Italy). Cells were grown in a 95% air, 5% CO_2_ atmosphere at 37 °C. In these conditions, the cell doubling time, Td, as determined from the growth curves, was 24 ± 1 h. 

### 2.2. Irradiation Procedure

For X-ray irradiation, cells were seeded in plates at least 48 h before treatment and irradiated at room temperature (RT) using a Gilardoni apparatus (250 kV, 6 mA, dose-rate 0.53 Gy/min). Unless otherwise indicated cells were irradiated with a dose of 4 Gy, then were trypsinized and seeded at the request density in fresh medium. Cells not irradiated were used as control in all the experiments and were trypsinized and seeded at the request density as irradiated cells.

### 2.3. Long-Term Proliferation Assessment

Irradiated and control cells were grown for 16 days with thee intermediate passages after 4, 8, and 12 days of culture. After harvesting cells were counted using a scepter handheld automated cell counter (Millipore, Burlington, MA, USA). The cumulative population doubling level (cPDL) after 4, 8, 12, and 16 days after X-rays treatment was calculated as the summation of PDs calculated with the formula: PDs = log2(N_f_/N_0_) where N_f_ is the final cell number and N_0_ is the initial number of seeded cells, in our case 100,000 cells per 25 cm^3^. The experiment was repeated two times.

### 2.4. Growth Curves 

After irradiation, cells were seeded at the density of 10^5^ cells; every 24 h cells were detached and counted with a Scepter handheld automated cell counter (Millipore) up to 168 h. Doubling time was determined as slope of the straight region of the growth fitted function. Results were obtained from four independent experiments. 

### 2.5. Flow Cytometry

For cell cycle analysis 1 × 10^6^ cells for each sample was washed twice with PBS, fixed dropwise with ice-cold ethanol (70%) and rehydrated with PBS. DNA staining was performed by incubating cells for 30 min at 37 °C in PBS containing 0.18 mg/mL propidium iodide (PI) and 0.4 mg/mL DNase-free RNase (type 1-A) (Sigma Aldrich, St. Louis, MO, USA). Samples were acquired with a Cytoflex (Beckman Coulter, Brea, CA, USA) equipped with a 488 nm laser source. Cell cycle analysis was performed using a Cytexpert v2.0 software. Doublets discrimination and exclusion were performed by an electronic gate on FL3-Area vs. FL3-Height. Each analysis was performed by acquiring 10,000 events/sample. Results were obtained from two independent experiments.

### 2.6. Collection of Chromosome Spreads 

Chromosome spreads were obtained following 30 min incubation in Calyculin-A (30 μM; Wako Chemicals, Japan). Only G_2_ condensed chromosomes have been scored in cytogenetic analysis. Prematurely condensed chromosomes (PCC) were collected by a standard procedure consisting of treatment with hypotonic solution (75 mM KCl) for 28 min at 37 °C, followed by fixation in freshly prepared Carnoy solution (3:1 *v/v* methanol/acetic acid). Cells were then seeded onto slides and utilized for cytogenetic analysis. 

### 2.7. Telomeric Quantitative FISH (Q-FISH) 

The Telomeric Quantitative FISH (Q-FISH) technique was performed as previously described by [37]. Images were captured with the M-search module of Metafer software (MetaSystems, Milan, Italy) at 63× magnification using an Axio Imager Z1 microscope (Zeiss, Jena, Germany) equipped with a Cool Cube 1 (CCD) camera (MetaSystems). Telomere size analysis was performed with the ISIS software (MetaSystems) that calculates telomere lengths as the ratio between the total telomere fluorescence (T) and the fluorescence of the centromeres of the two chromosomes (C), which is used as the internal reference in each metaphase spread analyzed and expressed as percentage (T/C%). At least 10 metaphases were analyzed for each sample in at least three independent experiments.

### 2.8. Intracellular Reactive Oxygen Species (ROS) Determination 

Cells were seeded at the density of 4 × 10^3^ inside 96-multiwell plates. Culture medium was discarded and a new medium containing 10 μM dichlorofluorescein 2′-7′-diacetate (DCFH-DA) (Sigma Aldrich) was added. Samples were incubated for 30 min in the dark, to allow the probe uptake. Cells were washed twice with PBS buffer and recovered for 30 min in the dark before analysis. DCFH-DA diffusion into cells was allowed by acetyl groups, while deacetylation by intracellular esterase activity prevented the DCFH exit from cells [38]. Emission analyses were performed by the automatic plate reader Victor 3V (Perkin Elmer, Waltham, MA, USA) and Wallac 1420 software. Excitation and emission wavelengths were set at 498 nm and 530 nm. The fluorescence intensity data obtained have been normalized for the cell number using Hoechst 33,342 at 350 nm as excitation and 461 nm as emission. To assess ROS content variations after X-ray exposure, cells were irradiated and analyzed at different times. For each sample analysis was repeated three times in at least two independent experiments.

### 2.9. N-acetylcysteine (NAC) Administration

ROS content variations were valuated even after N-acetylcysteine (NAC, Sigma Aldrich) antioxidant molecule administration. NAC was administrated 30 min prior and every 24 h after irradiations at the final concentration of 2 mM.

### 2.10. Telomere Dysfunction-Induced Foci (TIFs) Co-Immuno Staining

Cells were fixed with 4% paraformaldehyde (Sigma Aldrich), permeabilized with 0.2% Triton-X and blocked in PBS/BSA 1%. Samples were then co-immunostained over night at 4 °C, using a rabbit telomeric protein TRF1 antibody (Santa Cruz Biotechnology, Dallas, TX, USA) in combination with mouse yH2AX (Millipore) or a mouse 53BP1 antibody (Millipore). After washes in PBS/BSA1% samples were incubated with the secondary antibodies (anti-mouse Alexa 546 and anti-rabbit Alexa 488, respectively, Invitrogen, Carlsbad, CA, USA). Finally, slides were counterstained with DAPI and analyzed with fluorescence microscopy using an Axio-Imager Z1 microscope (Zeiss) equipped with the Metacyte module of the Metafer automated capture software and a CCD camera (MetaSystems). The frequency of foci and colocalization dots per cell were scored in 100 nuclei in at least two independent experiments.

### 2.11. Real Time Quantitative–Telomerase Repeat Amplification Protocol Assay (RTQ-TRAP)

Telomerase activity (TA) was measured by the SYBR green RTQ-TRAP assay, which was conducted as described elsewhere [39] with minor modifications. Briefly, the reaction was performed with protein extracts (1 × 10^3^ cells), 0.1 µg of telomerase primer TS, and 0.05 µg of anchored return primer ACX, in 25 µL of SYBR Green PCR Master Mix (Biorad, Hercules, CA, USA). The primer sequences were those reported by Kim and Wu (Kim and Wu, 1997). The reaction was performed using the Agilent AriaMx real-time PCR system (Agilent Technologies, Santa Clara, CA, USA), samples were incubated for 20 min at 25 °C and amplified in 35 PCR cycles with 30 s at 95 °C and 90 s at 60 °C (two step PCR). The threshold cycle values (Ct) were determined from semi-log amplification plots (log increase in fluorescence as a function of cycle number) and compared with standard curves generated from serial dilutions of telomerase-positive (tel+) U251MG cell extracts. HFFF2 heat-treated cells sample was obtained by boiling protein extract at 85 °C for 10 min. Telomerase activity was expressed relative to the telomerase-positive (tel+) sample. Each sample was analyzed in triplicate in at least two independent experiments. 

### 2.12. PML/RPA2-Telomere Immunofluorescence-FISH Staining

At different times after irradiation, cells were fixed for 20 min with 4% paraformaldehyde (Sigma Aldrich) in PBS at 4 °C, permeabilized with 0.2% Triton X-100 in PBS and blocked with PBS/BSA 1%. Cells were incubated with a rabbit polyclonal antibody against PML (H-238:sc5621, Santa Cruz Biotechnology) or a mouse mono-clonal anti-RPA2 antibody (Abcam, Cambridge, UK) overnight at 4 °C. After washing cells were incubated with Alexa 488 anti-rabbit antibody for PML or Alexa 488 anti-mouse antibody for RPA2 (Invitrogen). After immunostaining, telomeric FISH was performed as described above. Images were captured with fluorescence microscopy using an Axio-Imager Z1 microscope (Zeiss) equipped with the Metacyte module of the Metafer automated capture software and a CCD camera (MetaSystems). For PML analysis, a cell was considered positive when it showed at least three PML/telomere co-localization events. At least 50 nuclei in two independent experiments for PML and RPA2 were analyzed to identify events of possible co-localization. 

### 2.13. Chromosome Orientation-FISH (CO-FISH) Analysis 

After irradiation, cells were treated with 5′-bromo-2′-deoxyuridine (BrdU, Sigma Aldrich) at a final concentration of 2.5 × 10^−5^ M at 37° C for 24 h to allowed to replicate their DNA once. Chromosome spreads were prepared as described above. CO-FISH was performed as described previously [40] first using a (TTAGGG)3 probe labeled with FITC and then using a (CCCTAA)3 probe labeled with Cy3 (Panagene, Daejeon, South Korea). Images were captured with the M-search module of the Metafer software (MetaSystems) at 63× magnification using an Axio-Imager Z1 microscope (Zeiss) equipped with a CCD camera (MetaSystems) and analyzed by ISIS software (MetaSystems). T-SCEs were scored only when the double signals were visible with both the Cy3 and FITC probes. Experiments were repeated at least two times and 1000 chromosomes were analyzed for each sample. 

### 2.14. Whole Cell Extracts and Western Blotting 

Cells were harvested with trypsin, quickly washed in PBS, counted with a Scepter handheld automated cell counter (Millipore) and directly lysed in LDS sample buffer (Life Technologies, Carlsbad, CA, USA) at 10^4^ cells per μL. Proteins were gently homogenized using a 25-gauge syringe, denatured for 10 min at 70 °C and resolved by SDS-Page electrophoresis, transferred to nitrocellulose, blocked in 5% skim milk for 20 min and probed with the following primary antibodies: RAD51 (Santa Cruz, #sc-8349), ATRX (Santa Cruz, #sc-7152) and RPA2 (Abcam,#ab2175). HRP-linked anti-rabbit or anti-mouse (Amersham Pharmacia Biotech, Milano, Italy) secondary antibodies were used, and the HPR signal was visualized with Supersignal ECL substrate (Thermo Fisher Scientific, Waltham, MA, USA) following the manufacturer’s instructions. 

### 2.15. Chromatin Immunoprecipitation (ChIP) Assay and Telomere Dot-Blot

ChIP analysis was performed as previously described [41]. Briefly, 4 × 10^6^ cells were used for each experimental point. Chromatin fragments were incubated, overnight at 4 °C on a rotating platform with different antibodies: H3 (Abcam), H3K9me3 (Millipore), H4 (Abcam), H4K20me3 (Millipore) and the preimmune serum (Jackson Immuno Research Laboratories, Inc. Baltimore Pike, PA, USA). DNA was then recovered by phenol-chloroform extraction and ethanol precipitation, slot-blotted into a Hybond N^+^ membrane (Amersham Pharmacia Biotech) and hybridized with a telomeric probe (kindly provided by M. Blasco, Spanish National Cancer Research Centre-CNIO) obtained from a plasmid containing 1.6 kb of TTAGGG repeats labelled with α-32P. The signal was quantified using the ImageJ software. For total DNA samples, aliquots corresponding to a 1:10 dilution of the amount of lysate used in the immunoprecipitations were processed along with the rest of the samples during the crosslink reversal step. Data were normalized on the telomeric H3 and H4 signal, respectively. We represented the ChIP values as a percentage of the total input telomeric DNA, thus correcting for differences in the number of telomere repeats [42]. Experiments were performed at least two times.

### 2.16. Statistical Analysis

We performed the student’s *t*-test for the analysis of telomere length and for the density of telomeric marks in ChIP; we performed the one-way ANOVA test with Tukey’s post-test for the analysis of TIFs and APBs, for the analysis of telomeric signals in CO-FISH and for the analysis of the proteins. Significance was accepted for value *p <* 0.05.

## 3. Results

### 3.1. X-ray Irradiation Affects HFFF2 Proliferation.

Cell proliferation analysis in response to X-irradiation was performed by short- and long-term cell growth experiments.

**Figure 1:**
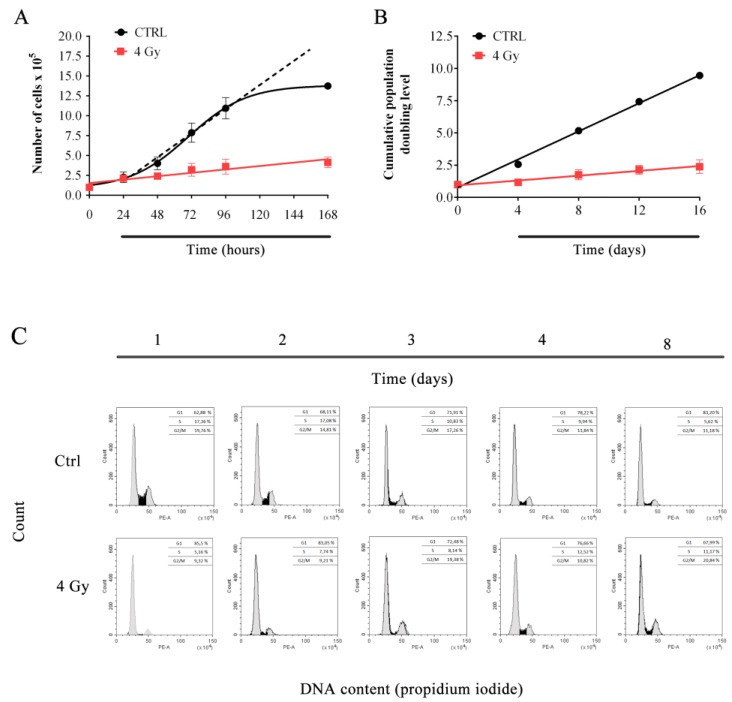
**Cell growth and cell cycle analysis**. Proliferation assay of control and irradiated HFFF2 cells performed by a short-term growth curve (**A**) from 0 to 168 h after irradiation, mean ± SEM (*N* = 4), and (**B**) long-term growth curve from 0 to 16 days after irradiation mean ± SD (*N* = 2). Error bars denote the standard deviation. (**C**) Representative monoparametric analysis of the DNA content in control and irradiated samples at 1, 2, 3, 4 and 8 days after X-ray exposure (*N* = 2).

Figure 1A shows short-term growth curves for X-ray- and control samples as evaluated in the first 168 h. As expected, data showed that X-rays were able to significantly affect cells proliferation, reducing cell growth by 70% at 96 h. Long-term (up to 16 days) growth curves allowed us to calculate cumulative population doubling level (cPDL) of irradiated and unirradiated cells (Figure 1B). Data indicated that irradiated cells continue to growth after irradiation even if, as expected, with a slower proliferation rate than sham irradiated samples (slope values are 0.54 ± 0.02 cPDL/time and 0.09 ± 0.01 cPDL/time for control and irradiated samples, respectively).

Cell cycle analysis was performed in order to further characterize short- and long-term (1–8 days) X-rays effect on cell proliferation. Data showed that, in control cells, S phase decreased (and G_1_ slightly increased) over-time as cell progressively reached confluency. On the other hand, first two days after irradiation, treated cells displayed S-phase and G_2_/M depletion. After day 2, S and G_2_/M slowly increased over-time, indicating that irradiated cells tried to reenter cell cycle (Figure 1C).

### 3.2. X-rays Induced Telomere Length Modulation Depends on the Level of IR-Induced OS

**Figure 2:**
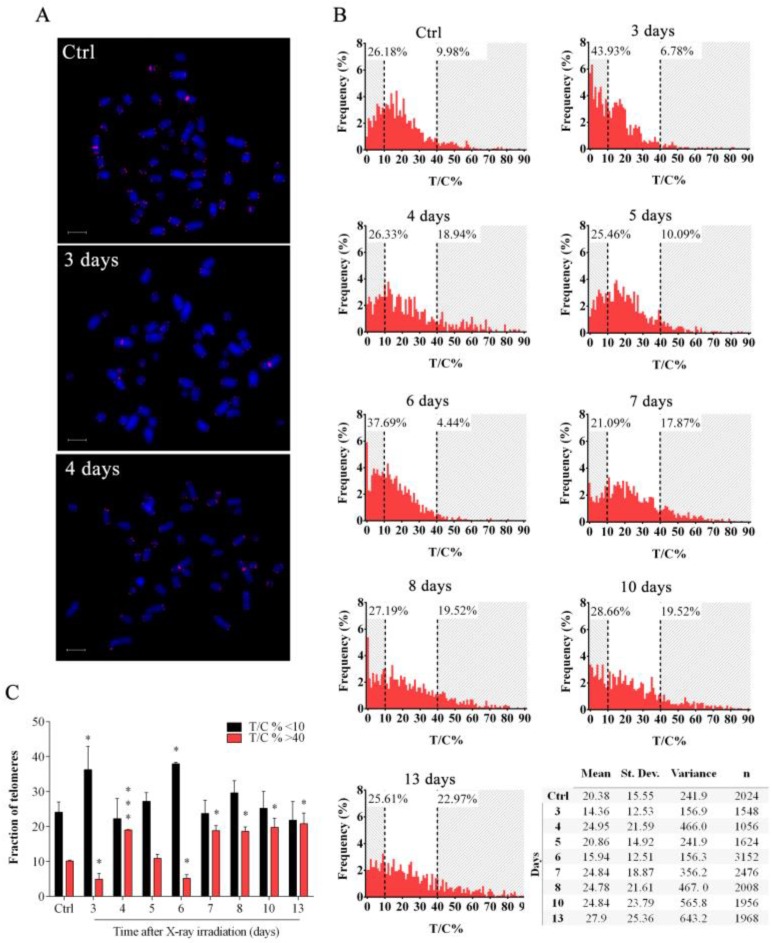
**Telomere length analysis in response to IR**. (**A**) Representative images of HFFF2 cells stained by Q-FISH at different times post X-ray exposure. Scale bar: 6 μm. (**B**) Telomere length distribution of control and irradiated samples at different days post X-ray exposure. Telomeres longer than 40 T/C% and shorter than 10 T/C% are highlighted in grey. The table shows the mean telomere lengths, the standard deviations, the variance and the number of telomeres analyzed for each sample. (**C**) Graphical representation of the fraction of telomere shorter than 10 T/C% (black columns) and longer than 40 T/C% (red columns). Data are expressed as mean values ± SD (*N* = 3). * *p* < 0.05; *** *p* < 0.001 by Student’s *t*-test.

We have previously shown that ionizing radiations can modulate telomere length over-time [37,43,44,45]. To characterize telomere length modulation after X-rays, we performed a detailed time-course analysis of telomere lengths (3–13 days) by centromere-calibrated Q-FISH (Figure 2A). Interestingly, we saw significant telomere length modulation displaying a complex pattern of periodic telomere shortening and lengthening over-time (Figure 2A,B). Shortly (three days) after the X-rays, we observed significant telomere erosion as shown by reduction of the fraction of longer telomeres and the increase of shorter telomeres. At day 4, a significant telomere lengthening (reduction of shorter telomere frequency and increase of longer telomere frequency) was observed, with telomere erosion at day 6 and once again lengthening that was stable between day 7 and day 13 (Figure 2C). This modulation of telomere length observed was consistent and statistically significant, suggesting the activation of a specific process that caused telomere length fluctuation over-time in the first 13 days after X-irradiation. In particular, our data indicated that after days 3 and 6, in which extensive telomere shortening occurs, a process was activated in order to reestablish normal telomere length. In addition, the heterogeneity of telomere lengths, evaluated through the calculation of variance (σ^2^) for each distribution plotted in Figure 2B, strongly increases at days 4, 7, 8, 10, and 13, and decreases at days 3 and 6 (Figure 2C). 

**Figure 3:**
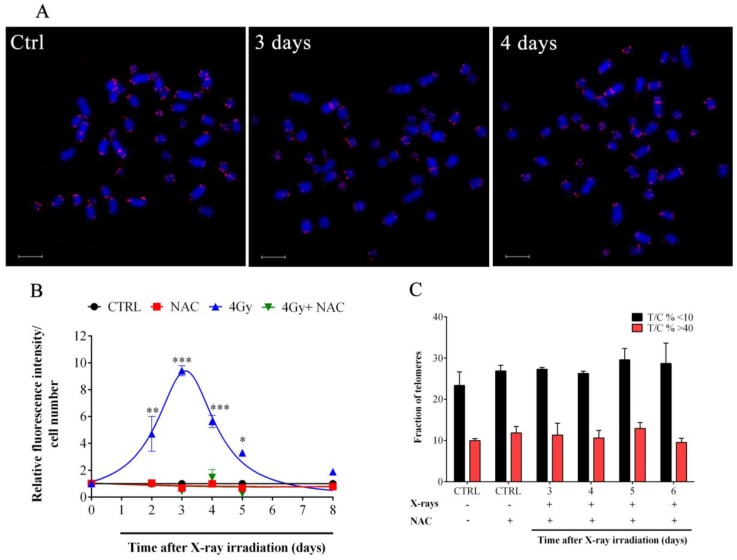
**NAC reduce IR-induced OS in HFFF2 fibroblasts**. (**A**) Representative images of HFFF2 cells treated with NAC and stained by Q-FISH at different times post X-ray exposure. Scale bar: 6 μm. (**B**) Measurement of the level of OS in control and irradiated samples treated or not with NAC by DCFH-DA assay. The values are expressed as mean values ± SD of the ratio between the relative fluorescence intensity and the cell number for each day analyzed (*N* = 2). * *p* < 0.05; ** *p* < 0.01; *** *p* < 0.001 by one-way ANOVA test with Tukey’s post-test. (**C**) The graph represents the fraction of telomeres shorter than 10 T/C% (black columns) and longer than 40 T/C% (red columns) treated (+) or not (-) with X-rays and NAC. Data are expressed as mean values ± SD (*N* = 2).

Telomeres are preferential targets for OS [46,47]. In order to determine if X-rays-induced OS may be the trigger of telomere length modulation observed (Figure 3A) we performed quantitative DCFH-DA measurement in the first 13 days after irradiation. Data in Figure 3B show that X-rays induced the maximum OS level after three days and subsequently OS decreased until day 8 when DCFH-DA values were similar to those detected in untreated controls (Figure 3B). In order to confirm the role of the IR-induced OS in the promotion of the telomere length modulation observed we performed experiments in the presence of the NAC, a well-known scavenger of ROS (Figure 3A). As shown in Figure 3B, NAC strongly reduced IR-induced DCFH-DA oxidation and hence fluorescence emission. Interestingly, telomere length modulation after X-ray exposure was not observed when cells were pretreated with NAC (Figure 3C), suggesting that oxidative damage is the major trigger of the telomere length modulation observed.

### 3.3. X-ray-Induced Telomere Damage Is Strictly Correlated to Telomere Shortening

**Figure 4:**
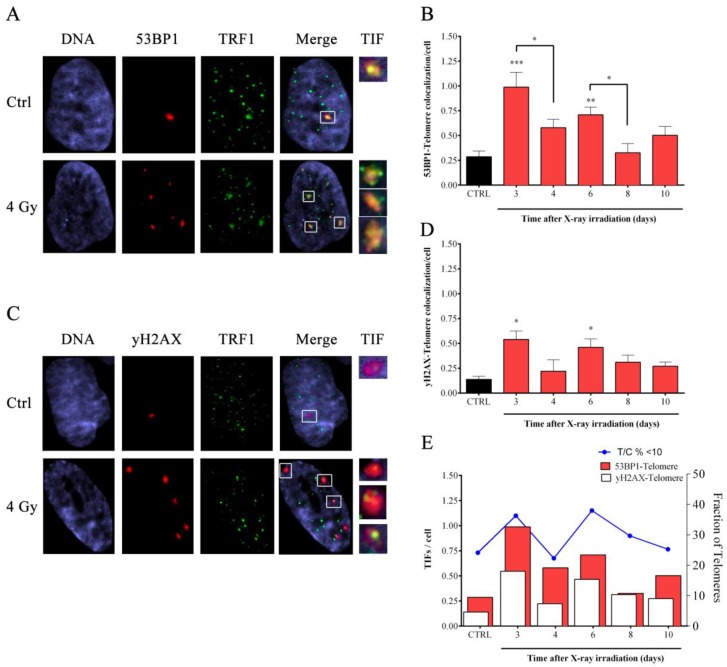
**Telomeric damage after X-rays exposure**. Images of HFFF2 treated with 4Gy of X-ray co-stained for 53BP1 (red spots) (**A**) or γH2AX (red spots) (**C**) and the telomeric protein TRF1 (green spots). TIF are enlarged (white boxes) to show the colocalization between telomeres (TRF1) and the DNA damage (53BP1 or γH2AX). (**B** and **D**) The graphs represent the colocalization frequency ± SD between TRF1 and 53BP1 (*N* = 2) (B) or TRF1 and γH2AX (*N* = 2) (**D**) in control and irradiated samples. (**E**) The graph shows the data obtained from TIF analysis (red and white bars for 53BP1 and γH2AX, respectively) and the fraction of telomere shorter than 10 T/C% (blue line). * *p* < 0.05; ** *p* < 0.01; *** *p* < 0.001 by one-way ANOVA test with Tukey’s post-test.

To test if telomere erosion observed resulted in telomere dysfunction, we monitored the accumulation of telomere dysfunction induced foci (TIFs) by co-immunostaining with antibodies against TRF1 and two different DNA damage markers (53BP1 and yH2AX) (Figure 4A,C).

For both the DNA damage markers, it appeared that X-rays induced significant telomere damage after three and six days from exposure, whereas values comparable to the untreated controls were observed at days 4, 8, and 10 (Figure 4B,D).

Interestingly, TIFs (from both TRF1-53BP1 and TRF1-yH2AX staining) appeared in association with telomere erosion at days 3 and 6 (Figure 4E). Indeed, the higher frequency of short telomeres corresponds to higher telomere damage observed whereas lower frequency of short telomeres is related to a lower yield of telomeric damage (Figure 4E).

### 3.4. IR-Induced Telomere Length Modulation Is Not Dependent on Telomerase Reactivation in Fibroblasts

**Figure 5:**
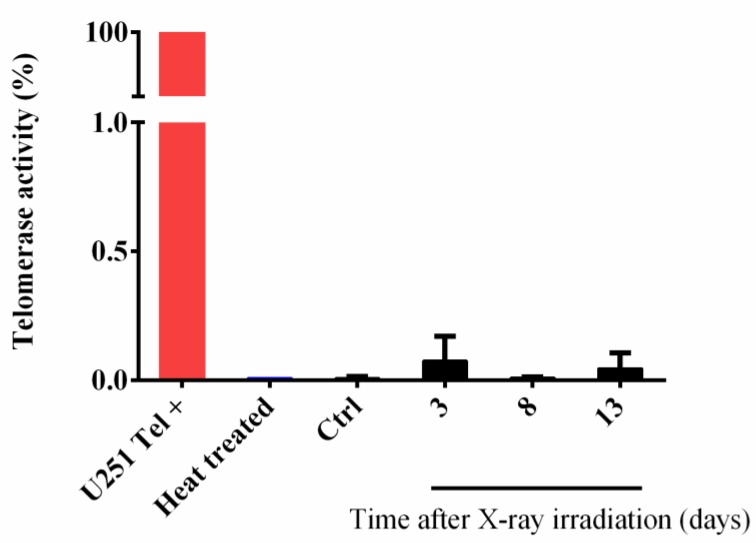
**Telomerase activity is not modulated by IR**. Telomerase activity in control and X-ray treated HFFF2 cells evaluated by RTQ-PCR TRAP assay. U251MG telomerase-positive cell line was used as positive control and a heat treated HFFF2 cell line was used as negative control. Values are expressed as mean values ± SD (*N* = 2).

To evaluate if telomere elongation observed at different times (3, 8 and 13 days) after X-ray treatment may be due to a telomerase-dependent mechanism, RTQ-PCR TRAP assay was performed to assess TA (Figure 5). A telomerase-positive glioma cell line (U251MG) was used as positive control and a heat-treated sample from HFFF2 cell line was used as negative control. The results did not show any change of TA in HFFF2 samples, excluding the participation of telomerase in the IR-induced telomere length modulation and pointing to a telomerase-independent mechanism.

### 3.5. Induction of APBs and TSCE in Response to X-rays Irradiation

**Figure 6:**
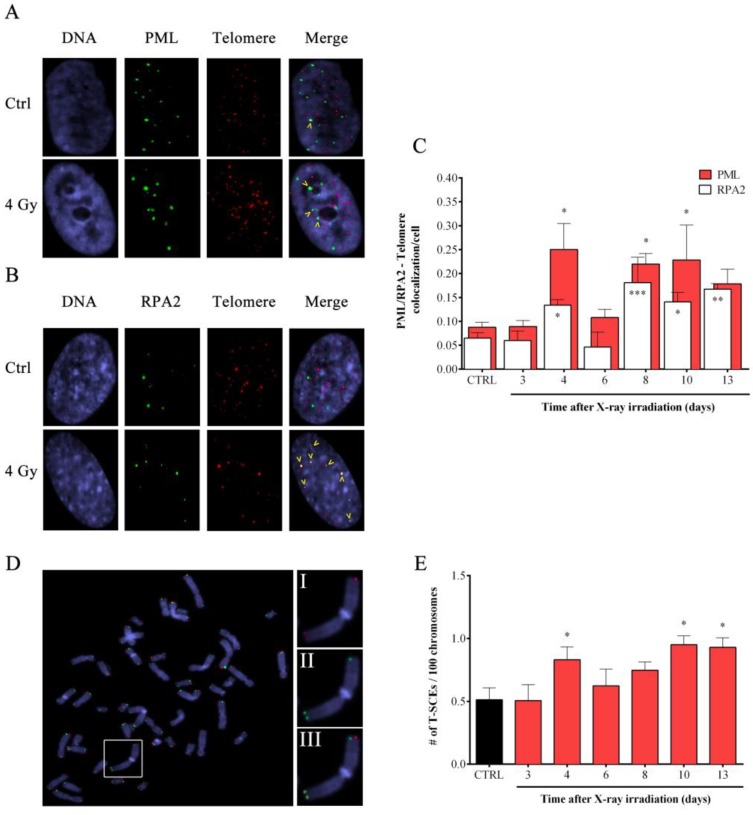
**Analysis of ALT hallmarks in HFFF2 cells after X-ray irradiation**. Images of control and 4Gy irradiated HFFF2 cells stained after 4 days for: (**A**) PML (green spots) and telomeres (red spots); (**B**) RPA2 (green spots) and telomeres (red spots). Colocalizations of PML (or RPA) with telomeres are highlighted with yellow ticks. (**C**) The graph represents the colocalization frequency of PML-telomere (red columns) and RPA2-telomere (white columns). Data are expressed as mean values ± SD (*N* = 2). (**D**) Representative image of a metaphase stained by CO-FISH. A chromosome (white box) enlarged shows both telomeres marked for both C- and G- probes; (I) red, (II) green and (III) merge. The frequency of the telomeric SCEs in control and irradiated samples are shown in figure (**E**), data are expressed as mean values ± SD (*N* = 2). * *p* < 0.05; ** *p* < 0.01; *** *p* < 0.001 by one-way ANOVA test with Tukey’s post-test.

To date, ALT is the only telomerase-independent telomere length maintenance mechanism. In order to evaluate the possible involvement of an ALT-like mechanism in response to telomeric damage induced by IR, we evaluated the presence of two largely accepted ALT hallmarks that are APBs and the T-SCEs**.** In particular, in Figure 6 are reported the data from immunoFISH experiments using antibodies against the PML or the RPA2 proteins in combination with a telomeric PNA probe (Figure 6A,B). For both, there was an increase in telomere colocalization dots per cells at 4, 8, 10 and 13 days after exposure to IR (Figure 6C). Basal colocalization frequencies in controls were about 0.08 and 0.06 dots/cell considering PML-telo and RPA2-telo experiments, respectively. Four days following X-ray irradiation, the number of co-localizations increased to 0.25 and 0.13 before returning to basal levels at day 6 (0.10 and 0.04). However, from day 8 to 13, colocalization frequencies increased again with values between 0.2 and 0.24 for PML-telo and 0.12 and 0.16 for RPA2-telo experiments (Figure 6C).

Next, we performed CO-FISH experiments in samples exposed to IR in order to determine the presence of telomere recombination (Figure 6D). CO-FISH analysis indicated a significant induction of T-SCEs after 4, 10 and 13 days after IR exposure. Basal levels of T-SCEs were ~0.5 per 100 chromosomes which increased to 0.85 at four days following IR exposure. As reported for APBs, after a first induction observed at 4 days, we saw a return to basal values (at day 6 and 8) followed by another increase between day 10 and 13 (frequencies comprised 0.8 and 1.0) (Figure 6E). Taken together, these data suggest the involvement of a telomere recombination process in response to telomeric damage induced by IR in HFFF2 human primary fibroblasts. 

### 3.6. IR-Dependent Induction of Homologous Recombination Proteins RAD51 and RPA2 Was Coupled to the Suppression of the Chromatin-Remodeling Factor ATRX 

**Figure 7:**
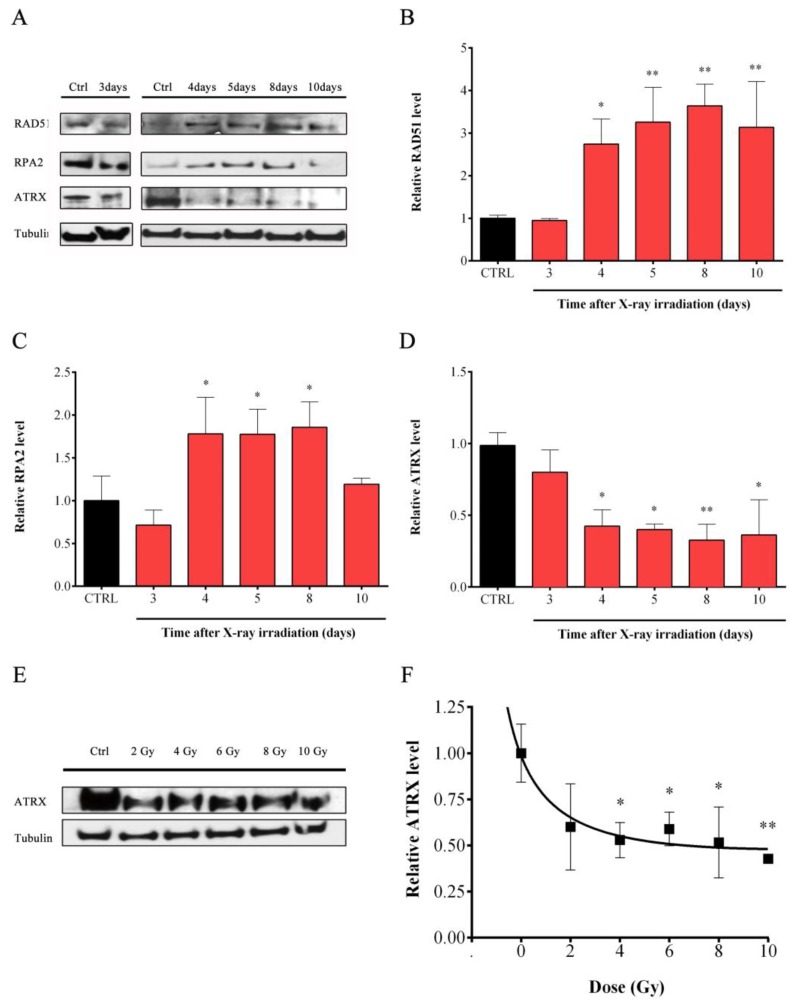
**X-rays induced modulation of proteins involved in ALT**. (**A**) Representative western immunoblotting showing RAD51, RPA and ATRX proteins amount in control and 4 Gy-irradiated samples harvested at different days post exposure. Tubulin was used to ensure the equal proteins loading. The quantification of the amount of the proteins RAD51 (**B**), RPA (**C**) and ATRX (**D**) in control and irradiated cells are expressed as mean values ± SD (*N* = 2 for RAD51 and ATRX; *N* = 3 for RPA). (**E**) Representative western immunoblotting showing ATRX protein level in control and irradiated samples (dose ranging from 2 to 10 Gy). (**F**) Quantification of the ATRX protein amount in cells irradiated at different doses of X-rays and are expressed as mean values ± SD (*N* = 3). * *p* < 0.05; ** *p* < 0.01 by one-way ANOVA test with Tukey’s post-test.

To gain further insights into the IR-induced telomeric HR-mediated mechanism observed, we analyzed proteins that are modulated in cancer cells that activate ALT telomere maintenance. Interestingly, the expression of both RAD51 and RPA2, two well-known HR proteins, were significantly induced by IR from day 4 until days 8 and 10, respectively (Figure 7A). At day 8, the maximum increase of both proteins, 3.5-fold increase for RAD51 and a two-fold increase for RPA2, was observed (Figure 7B,C).

This increase in RAD51 and RPA2 correlated with a strong IR-dependent reduction of the remodeling factor ATRX (Figure 7A). Time course analysis of ATRX protein levels indicated that a significant reduction of ATRX was observed after 4 days (about 60% reduction) and subsequently, ATRX levels remained reduced until day 10 (Figure 7D). To further confirm this IR induced changes in ATRX levels, we assessed how different doses of IR could affect ATRX (Figure 7E). Data indicated that a dose of X-rays greater than 4 Gy was sufficient to reduced ATRX protein levels. A reduction was also observed at 2 Gy but data was not significant (Figure 7F). 

### 3.7. Reduction of Telomeric Histone Modification Mark in Correspondence to Telomeric HR Activation

**Figure 8:**
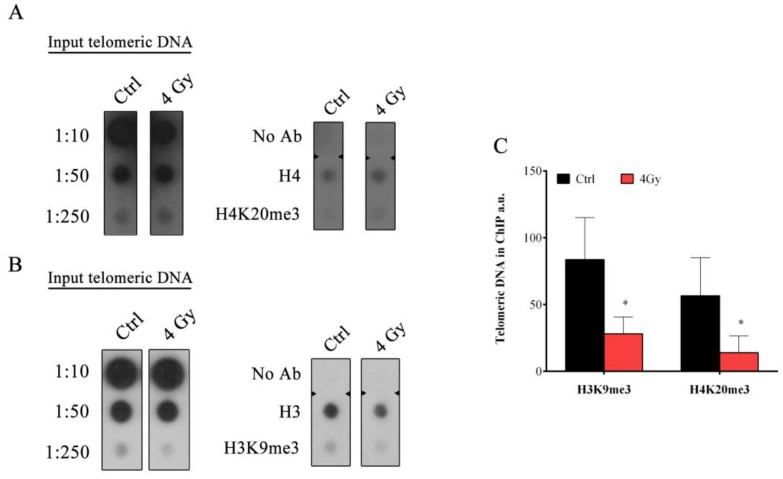
Chromatin immunoprecipitation and telomere dot-blot for H3K9me3 and H4K20me3. (**A**-**B**) Representative blot of a ChIP experiments showing samples from control and 4 Gy-irradiated HFFF2 cells. Telomeric chromatin was immunostained with the indicated antibody (H3, H3K9me3, H4 and H4K20me3) (No Ab—No Antibody). Telomeric H3 and H4 signals were used for quantification. For this analysis, a 1:250 input ratio was used. (**C**) Quantification of immunoprecipitated telomeric sequences performed after normalization to the telomeric H3 and H4 signal (*N* = 2). The data are expressed as a percentage of the total telomeric DNA in arbitrary units (a.u) ± SD. * *p* < 0.05 by Student’s *t*-test.

Considering recent data about the correlation between ATRX and epigenetic changes in ALT cells [48], and taking into account our observation about the modulation of ATRX in response to IR, we performed the ChIP assay, specifically at telomeres (Figure 8). In order to investigate the telomeric chromatin status four days after X-ray irradiation, at which telomere modulation and ALT hallmarks (APBs and TSCE) are evident, we chose to monitor two characteristics of telomeric heterochromatin: H3K9me3 and H4K20me3. The quantification of immunoprecipitated telomeric DNA with H3K9me3 and H4K20me3 modifications was performed after normalization to the telomeric H3 and H4 signal respectively (Figure 8A,B). The results obtained showed a significant reduction for both H3K9me3 (from 83.57% in the control sample to 28.1% in 4 Gy-irradiated sample) and H4K20me3 (from 56.6% to 14% respectively) (Figure 8C). 

## 4. Discussion

The role of the ALT pathway in normal cells has been recently debated and new evidences indicate that HR-mediated telomere maintenance/repair may not be exclusive to cancer cells [37,49,50]. Indeed, in yeast short telomeres can stimulate telomere recombination, perhaps due to the loss of telomere capping [51] as well as normal primary murine cells harboring very short telomeres [49]. In addition, it has been proposed that cycling human fibroblasts are able to repair telomeric DSBs by HR and that siRNA mediated or chemical inhibition of *RAD51* impairs telomeric recombination [16]. In accordance with previous published data [37], the present work reports that exposure to X-rays can induce telomere length changes and telomere recombination in human primary fibroblasts. This correlated with the transient appearance of features considered hallmarks of ALT cancer cells. A detailed Q-FISH analysis indicated that after exposure to X-rays, mean telomere lengths change in a time-dependent manner, with two consecutive rounds of telomere shortening followed by telomere elongations. Interestingly, the analysis of short (or long) telomere fractions allowed us to appreciate telomere length variations that are suggestive of a rapid process that alters telomere maintenance mechanisms and is probably activated in response to telomeric damage. Indeed, IR is a kind of genotoxic stress capable to induce DSBs in a stochastic manner throughout the genome. The possibility that IR directly induces a subset of lesions in telomeric DNA is very questionable due to the negligible fraction of telomeric DNA in comparison to genomic DNA (0.02%), however some authors proposed that while most DNA damage is repaired, telomeres resist repair and the fraction of persisting DDR foci at telomeres progressively increases over time [52]. Nonetheless, it is well known that ionizing radiation, such as X-rays, principally induce DSBs through an (OS-mediated) indirect mechanism [53]. Several papers in the literature indicated that OS specifically target G-rich regions such as telomeres [54], determining the occurrence of 8-oxo-Guanine (8-oxoG) that in turn increases the rate of stalling of replication forks at telomeres [41,55]. Remarkably, the measurement of the OS was significantly higher in samples exposed to 4Gy of X-rays than in mock-irradiated controls and persisted until day 5 after irradiation. This is in agreement with reported data from earlier studies using normal and immortalized human fibroblasts [56,57,58] and suggests that IR-induced OS may represent the trigger of telomeric damage observed in the first days after treatment. To confirm this hypothesis, we showed that the pretreatment of IR-exposed fibroblasts with the ROS-scavenging agent NAC, a well-known radioprotective compound, reduced oxidative stress to control levels and totally abrogated telomere lengths variations, strongly suggesting that IR induces telomere damage through an OS-dependent mechanism. To directly determine the capability of IR to induce DNA damage in telomeric regions, analysis of TIFs was performed after X-rays exposure. The colocalization of the telomeric protein TRF1 with γ-H2AX and 53BP1 showed that telomeric damage was induced by irradiation in agreement with previous findings in which IR (but also OS) induced telomere erosion and telomere-positive anaphase bridges or nucleoplasmic bridges [45,54]. Notably, considering the time frame analyzed (3–13 days) our data indicated that the amount of telomeric damage was significantly higher compared to control at days 3 and 6 post-irradiation, in which we observed the highest yield of telomere shortening. In a previous study, we showed that exposure to high-linear energy transfer (LET) IR can activate a transient form of the ALT pathway in HFFF2 human primary fibroblasts [37], indicating for the first time that IR-induced DNA damage may activate telomeric recombination. The ALT activation determined telomere lengthening and was supported by the induction of a number of ALT-associated hallmarks [37]. It is becoming increasingly evident that damage at telomeres might favor ALT activation in either telomerase-positive cancer cells or in primary cells promoting telomere recombination [49,59,60]. Though it is widely accepted that telomeric HR is active in ALT cells, the precise molecular basis for this predisposition is unclear. It might be due to loss of *ATRX* expression that fundamentally alters chromatin assembly due the absence of its chromatin remodeling activity. Indeed, we do observe a strong reduction in *ATRX* expression following x-ray irradiation. Another possibility is that the binding of shelterin constituents like TRF2 is transiently disrupted in response to X-rays [41,61,62]. In addition, there is evidence that as telomeres elongate beyond normal limits the binding of TRF1 and TRF2 become diluted [48]. This has the dual consequences of altering telomere protection and also creates potential binding sites for other DNA binding factors (e.g., Orphan Nuclear Receptor proteins and TZAP). In this respect, it is sensitive to speculate that DNA damage at telomeres may preferentially activate HR-mediated repair due to both its intrinsic nature of tandem repeated sequence and also to the strong downregulation of NHEJ by TRF2 [12]. ATM damage signaling by ATM is primarily repressed at telomeres by TRF2 [63,64] and in general DSB repair at telomeres is normally repressed by the action of shelterin proteins such as TRF2 and POT1. In particular, NHEJ is strongly inhibited due to the very deleterious outcome of telomeric rejoining and consequent chromosome end-to-end fusions [65]. Less is known about HR repression that seems to rely (at least in part) on ATRX inhibition of RPA loading at telomeres [66] and on ATRX-DAXX ability to maintain compact chromatin status at telomeres. Indeed, reintroducing ATRX into ALT cells suppresses T-SCEs, APBs, c-circles formation, and inter-chromosomal telomeric recombination [29,67], indicating that HR permissive environment may depend on alteration of chromatin status [65]. In this regard, the transient activation of an ALT-like pathway in response to IR may be interpreted as a DNA damage response of the cell to telomere damage in accordance with recent evidence demonstrating that also normal cells, such as fibroblasts, activate telomeric HR-mediated repair to cope with telomere damage as demonstrated by the induction of some specific ALT hallmarks such as telomere-clustering and T-SCEs [16]. 

To confirm whether transient ALT/telomeric HR-repair is involved in the processing of X-rays-induced DNA damage at telomeres, we checked some of the hallmarks used to define ALT in cancer cells such as TSCE and colocalization of PML (or RPA2) with telomeric DNA. We found that T-SCE frequency and both PML and RPA2 colocalization with telomeric DNA increased shortly after telomere erosion/damage (within 24 h from telomere erosion, and in particular at day 4 and day 8). 

Remarkably, the time-course analysis allowed us to observe a dampened oscillatory behavior for all the endpoints evaluated. Initially we observed telomere shortening and DNA damage at telomeres that activate telomeric recombination, as shown by colocalization of PML and telomere and T-SCE induction, leading to fast telomere lengthening. After that, a second round of telomere shortening and damage was observed, probably determined by the persistence of oxidative stress. Telomeric damage activated again telomeric recombination and telomere lengthening, inducing another round of DNA damage and response at telomeres, although with a lower amplitude than the first one.

Our data strongly point out the activation of a recombination mediated telomeric repair pathway (that shares features with the ALT pathway) in response to IR-induced OS. This is consistent with a previous published study in which we demonstrated the activation of telomere recombination in response to chronic exposure to H_2_O_2_ [68].

Other than telomeric recombination and localization of telomeric DNA in the PML bodies, also telomere proteins involved in HR (and upregulated in ALT) such as RAD51 and RPA2 were strongly modulated. Previously published papers revealed that *RAD51* was upregulated in response to IR in the first hours after irradiations in glioma cells [69] and low dose gamma-irradiation induce *RAD51* expression through increased oxidative stress, histone deacetylation and reduction of miR-193b-3p [70]. Our data point in the same direction indicating a RAD51 increase starting from day 4 after IR when we also observed levels of OS higher than those of sham-irradiated controls. Also RPA2 was increased starting for day 4 after IR supporting the activation of HR. Interestingly, also ATRX, a chromatin remodeling factor strongly downregulated in ALT cancer cells [26,27], was modulated in response to IR. As far as we know, only a study performed on human esophageal cancer cells has highlighted an IR-dependent modulation of ATRX [71] shortly after irradiation. Interestingly, our data indicated that ATRX was downregulated starting from day 4 and at least till day 10 at which protein levels remained significantly lower than those of sham-irradiated controls. Moreover, to further confirm the IR-dependent modulation of ATRX we also performed a dose-response experiment at day 4 after IR. Data showed an appreciable reduction of the protein level in response to 2 Gy and significant reduction was found starting from 4 Gy of X-rays. Doses higher than 4 Gy did not seem to further reduce ATRX protein level. ATRX downregulation is very often seen in ALT cancer cells, and current knowledge indicates that ATRX depletion may be linked to epigenetic changes (such as the reduction of the trimethylated form of both H3K9 and H4K20) in telomeric regions that favor chromatin relaxation and thus the onset of HR [48]. According to this view, at day 4 after irradiation we found a significant reduction of both H3K9 and H4K20 trimethylated forms, indicating that, besides the reduction of ATRX, epigenetic changes occur in order to favor DNA repair mechanisms but, specifically at telomeres, this may be an additional feature that facilitates the onset of HR mediated repair and telomere length modulation observed. ATRX governs also the cell cycle dependent regulation of the telomeric non-coding RNA TERRA and its reduction determines the persistent high level of TERRA in G2/M phase and the increase of RPA at telomeres [66]. Interestingly, TERRA can also be induced by DNA damage in a p53-dependent manner [72]. This is consistent with the idea that relaxation of telomeric DNA makes it more accessible to DNA modifying proteins, nucleases [73,74] and other enzymes required for efficient HR. In a similar manner, torsional stress is relieved when DSBs are induced in chromosomal DNA [75,76] as in the case of IR-exposed samples. We hypothesize that IR determines the activation of a transient ALT-like pathway triggered by telomeric DNA damage in a contest of chromatin relaxation with the aim to repair lesions in telomeric DNA, determining the rapid increase of telomere length, especially at very short telomeres. Remarkably, the process repeats until DNA damage was sustained at telomeres with a dampened oscillatory behavior. 

Altogether, these data indicated for the first time that IR-induced damage at telomeres activates telomeric recombination as telomeric DNA damage response and that the process may be sustained if telomeric DNA damage persists, contributing to clarify the role of HR-mediated repair at telomeres and highlight the strong similarities between such repair mechanism and ALT. Our work, and recent evidence coming from the literature, point to the physiological, transient activation of HR-based DNA damage repair at telomeres, sharing several features with ALT as it is characterized in cancer cells. Defining the differences between a transient stress-induced telomeric repair pathway based on HR and the ALT observed in cancer cells is not easy, and the latter may probably be considered as a completely deregulated and over functioning form of the former. Nonetheless, we firmly think that the characterization of HR-mediated telomere repair in normal human primary cells may contribute to the understanding of ALT, and may facilitate the identification of ALT-specific proteins in the light of a future therapeutic targeting of this telomere maintenance mechanism.

## Abbreviations

ALT	Alternative lengthening of telomeres
IR	Ionizing radiations
HR	Homologous recombination
DSB	Double strand break
NHEJ	Non-homologous end-joining
T-SCE	Telomere-sister chromatid exchange
ECTR	Extrachromosomal telomeric repeats
PML	Promyelocytic leukemia protein
APB	ALT-associated PML (promyelocytic leukemia protein) body
DDR	DNA damage response
OS	Oxidative stress
CPDL	Cumulative population doubling level
TIF	Telomere dysfunction-induced foci
PCC	Prematurely condensed chromosomes

## References

[1] Pandita T.K., Pathak S., Geard C.R. (1995). Chromosome end associations, telomeres and telomerase activity in ataxia telangiectasia cells. Cytogenet. Cell Genet..

[2] Blackburn E.H. (2001). Switching and signaling at the telomere. Cell.

[3] de Lange T. (2005). Shelterin: The protein complex that shapes and safeguards human telomeres. Genes Dev..

[4] Tommerup H., Dousmanis A., de Lange T. (1994). Unusual chromatin in human telomeres. Mol. Cell. Biol..

[5] Lejnine S., Makarov V.L., Langmore J.P. (1995). Conserved nucleoprotein structure at the ends of vertebrate and invertebrate chromosomes. Proc. Natl. Acad. Sci. USA.

[6] Nikitina T., Woodcock C.L. (2004). Closed chromatin loops at the ends of chromosomes. J. Cell Biol..

[7] Broccoli D., Smogorzewska A., Chong L., de Lange T. (1997). Human telomeres contain two distinct Myb-related proteins, TRF1 and TRF2. Nat. Genet..

[8] de Lange T. (2002). Protection of mammalian telomeres. Oncogene.

[9] Smogorzewska A., de Lange T. (2004). Regulation of telomerase by telomeric proteins. Annu. Rev. Biochem..

[10] Blasco M.A. (2007). The epigenetic regulation of mammalian telomeres. Nat. Rev. Genet..

[11] van Steensel B., Smogorzewska A., de Lange T. (1998). TRF2 protects human telomeres from end-to-end fusions. Cell.

[12] Karlseder J., Broccoli D., Dai Y., Hardy S., de Lange T. (1999). p53- and ATM-dependent apoptosis induced by telomeres lacking TRF2. Science.

[13] Sfeir A., de Lange T. (2012). Removal of shelterin reveals the telomere end-protection problem. Science.

[14] Kruk P.A., Rampino N.J., Bohr V.A. (1995). DNA damage and repair in telomeres: Relation to aging. Proc. Natl. Acad. Sci. USA.

[15] Petersen S., Saretzki G., von Zglinicki T. (1998). Preferential accumulation of single-stranded regions in telomeres of human fibroblasts. Exp. Cell Res..

[16] Mao P., Liu J., Zhang Z., Zhang H., Liu H., Gao S., Rong Y.S., Zhao Y. (2016). Homologous recombination-dependent repair of telomeric DSBs in proliferating human cells. Nat. Commun..

[17] Oikawa S., Tada-Oikawa S., Kawanishi S. (2001). Site-specific DNA damage at the GGG sequence by UVA involves acceleration of telomere shortening. Biochemistry.

[18] Londono-Vallejo J.A., Der-Sarkissian H., Cazes L., Bacchetti S., Reddel R.R. (2004). Alternative lengthening of telomeres is characterized by high rates of telomeric exchange. Cancer Res..

[19] Cesare A.J., Reddel R.R. (2010). Alternative lengthening of telomeres: Models, mechanisms and implications. Nat. Rev. Genet..

[20] Murnane J.P., Sabatier L., Marder B.A., Morgan W.F. (1994). Telomere dynamics in an immortal human cell line. EMBO J..

[21] Bryan T.M., Englezou A., Gupta J., Bacchetti S., Reddel R.R. (1995). Telomere elongation in immortal human cells without detectable telomerase activity. EMBO J..

[22] Cesare A.J., Griffith J.D. (2004). Telomeric DNA in ALT cells is characterized by free telomeric circles and heterogeneous t-loops. Mol. Cell. Biol..

[23] Nabetani A., Ishikawa F. (2009). Unusual telomeric DNAs in human telomerase-negative immortalized cells. Mol. Cell. Biol..

[24] Yeager T.R., Neumann A.A., Englezou A., Huschtscha L.I., Noble J.R., Reddel R.R. (1999). Telomerase-negative immortalized human cells contain a novel type of promyelocytic leukemia (PML) body. Cancer Res..

[25] Tang J., Wu S., Liu H., Stratt R., Barak O.G., Shiekhattar R., Picketts D.J., Yang X. (2004). A novel transcription regulatory complex containing death domain-associated protein and the ATR-X syndrome protein. J. Biol. Chem..

[26] Heaphy C.M., de Wilde R.F., Jiao Y., Klein A.P., Edil B.H., Shi C., Bettegowda C., Rodriguez F.J., Eberhart C.G., Hebbar S. (2011). Altered telomeres in tumors with ATRX and DAXX mutations. Science.

[27] Lovejoy C.A., Li W., Reisenweber S., Thongthip S., Bruno J., de Lange T., De S., Petrini J.H., Sung P.A., Jasin M. (2012). Loss of ATRX, genome instability, and an altered DNA damage response are hallmarks of the alternative lengthening of telomeres pathway. PloS Genet..

[28] Schwartzentruber J., Korshunov A., Liu X.Y., Jones D.T., Pfaff E., Jacob K., Sturm D., Fontebasso A.M., Quang D.A., Tonjes M. (2012). Driver mutations in histone H3.3 and chromatin remodelling genes in paediatric glioblastoma. Nature.

[29] Napier C.E., Huschtscha L.I., Harvey A., Bower K., Noble J.R., Hendrickson E.A., Reddel R.R. (2015). ATRX represses alternative lengthening of telomeres. Oncotarget.

[30] Tagami H., Ray-Gallet D., Almouzni G., Nakatani Y. (2004). Histone H3.1 and H3.3 complexes mediate nucleosome assembly pathways dependent or independent of DNA synthesis. Cell.

[31] O’Sullivan R.J., Almouzni G. (2014). Assembly of telomeric chromatin to create ALTernative endings. Trends Cell Biol..

[32] O’Sullivan R.J., Kubicek S., Schreiber S.L., Karlseder J. (2010). Reduced histone biosynthesis and chromatin changes arising from a damage signal at telomeres. Nat. Struct. Mol. Biol..

[33] Dilley R.L., Verma P., Cho N.W., Winters H.D., Wondisford A.R., Greenberg R.A. (2016). Break-induced telomere synthesis underlies alternative telomere maintenance. Nature.

[34] Sobinoff A.P., Pickett H.A. (2017). Alternative Lengthening of Telomeres: DNA Repair Pathways Converge. Trends Genet..

[35] Doksani Y., de Lange T. (2016). Telomere-Internal Double-Strand Breaks Are Repaired by Homologous Recombination and PARP1/Lig3-Dependent End-Joining. Cell Rep..

[36] Cho N.W., Dilley R.L., Lampson M.A., Greenberg R.A. (2014). Interchromosomal homology searches drive directional ALT telomere movement and synapsis. Cell.

[37] Berardinelli F., Antoccia A., Cherubini R., De Nadal V., Gerardi S., Cirrone G.A., Tanzarella C., Sgura A. (2010). Transient activation of the ALT pathway in human primary fibroblasts exposed to high-LET radiation. Radiat. Res..

[38] Gnocchi D., Leoni S., Incerpi S., Bruscalupi G. (2012). 3,5,3′-triiodothyronine (T3) stimulates cell proliferation through the activation of the PI3K/Akt pathway and reactive oxygen species (ROS) production in chick embryo hepatocytes. Steroids.

[39] Wege H., Chui M.S., Le H.T., Tran J.M., Zern M.A. (2003). SYBR Green real-time telomeric repeat amplification protocol for the rapid quantification of telomerase activity. Nucleic Acids Res..

[40] Bailey S.M., Goodwin E.H., Cornforth M.N. (2004). Strand-specific fluorescence in situ hybridization: The CO-FISH family. Cytogenet. Genome Res..

[41] Coluzzi E., Leone S., Sgura A. (2019). Oxidative Stress Induces Telomere Dysfunction and Senescence by Replication Fork Arrest. Cells.

[42] Benetti R., Garcia-Cao M., Blasco M.A. (2007). Telomere length regulates the epigenetic status of mammalian telomeres and subtelomeres. Nat. Genet..

[43] Sgura A., Antoccia A., Berardinelli F., Cherubini R., Gerardi S., Zilio C., Tanzarella C. (2006). Telomere length in mammalian cells exposed to low- and high-LET radiations. Radiat. Prot. Dosim..

[44] Berardinelli F., Nieri D., Sgura A., Tanzarella C., Antoccia A. (2012). Telomere loss, not average telomere length, confers radiosensitivity to TK6-irradiated cells. Mutat. Res..

[45] Berardinelli F., Antoccia A., Buonsante R., Gerardi S., Cherubini R., De Nadal V., Tanzarella C., Sgura A. (2013). The role of telomere length modulation in delayed chromosome instability induced by ionizing radiation in human primary fibroblasts. Environ. Mol. Mutagenesis.

[46] Opresko P.L., Fan J., Danzy S., Wilson D.M., Bohr V.A. (2005). Oxidative damage in telomeric DNA disrupts recognition by TRF1 and TRF2. Nucleic Acids Res..

[47] von Zglinicki T. (2000). Role of oxidative stress in telomere length regulation and replicative senescence. Ann. N. Y. Acad. Sci..

[48] Conomos D., Pickett H.A., Reddel R.R. (2013). Alternative lengthening of telomeres: Remodeling the telomere architecture. Front. Oncol..

[49] Morrish T.A., Greider C.W. (2009). Short telomeres initiate telomere recombination in primary and tumor cells. PloS Genet..

[50] Neumann A.A., Watson C.M., Noble J.R., Pickett H.A., Tam P.P., Reddel R.R. (2013). Alternative lengthening of telomeres in normal mammalian somatic cells. Genes Dev..

[51] McEachern M.J., Haber J.E. (2006). Break-induced replication and recombinational telomere elongation in yeast. Annu. Rev. Biochem..

[52] Fumagalli M., Rossiello F., Clerici M., Barozzi S., Cittaro D., Kaplunov J.M., Bucci G., Dobreva M., Matti V., Beausejour C.M. (2012). Telomeric DNA damage is irreparable and causes persistent DNA-damage-response activation. Nat. Cell Biol..

[53] Ward J.F. (1998). Nature of Lesions Formed by Ionizing Radiation.

[54] Coluzzi E., Colamartino M., Cozzi R., Leone S., Meneghini C., O’Callaghan N., Sgura A. (2014). Oxidative stress induces persistent telomeric DNA damage responsible for nuclear morphology change in mammalian cells. PloS ONE.

[55] von Zglinicki T., Pilger R., Sitte N. (2000). Accumulation of single-strand breaks is the major cause of telomere shortening in human fibroblasts. Free Radic. Biol. Med..

[56] Rugo R.E., Secretan M.B., Schiestl R.H. (2002). X radiation causes a persistent induction of reactive oxygen species and a delayed reinduction of TP53 in normal human diploid fibroblasts. Radiat. Res..

[57] Kobashigawa S., Suzuki K., Yamashita S. (2011). Ionizing radiation accelerates Drp1-dependent mitochondrial fission, which involves delayed mitochondrial reactive oxygen species production in normal human fibroblast-like cells. Biochem. Biophys. Res. Commun..

[58] Dettmering T., Zahnreich S., Colindres-Rojas M., Durante M., Taucher-Scholz G., Fournier C. (2015). Increased effectiveness of carbon ions in the production of reactive oxygen species in normal human fibroblasts. J. Radiat. Res..

[59] Hu Y., Shi G., Zhang L., Li F., Jiang Y., Jiang S., Ma W., Zhao Y., Songyang Z., Huang J. (2016). Switch telomerase to ALT mechanism by inducing telomeric DNA damages and dysfunction of ATRX and DAXX. Sci. Rep..

[60] Liu H., Xie Y., Zhang Z., Mao P., Liu J., Ma W., Zhao Y. (2018). Telomeric Recombination Induced by DNA Damage Results in Telomere Extension and Length Heterogeneity. Neoplasia.

[61] Tanaka H., Mendonca M.S., Bradshaw P.S., Hoelz D.J., Malkas L.H., Meyn M.S., Gilley D. (2005). DNA damage-induced phosphorylation of the human telomere-associated protein TRF2. Proc. Natl. Acad. Sci. USA.

[62] Huda N., Tanaka H., Mendonca M.S., Gilley D. (2009). DNA damage-induced phosphorylation of TRF2 is required for the fast pathway of DNA double-strand break repair. Mol. Cell. Biol..

[63] Karlseder J., Hoke K., Mirzoeva O.K., Bakkenist C., Kastan M.B., Petrini J.H., de Lange T. (2004). The telomeric protein TRF2 binds the ATM kinase and can inhibit the ATM-dependent DNA damage response. PloS Biol..

[64] Denchi E.L., de Lange T. (2007). Protection of telomeres through independent control of ATM and ATR by TRF2 and POT1. Nature.

[65] Sfeir A., Denchi E.L. (2016). Stressed telomeres without POT1 enhance tumorigenesis. Oncotarget.

[66] Flynn R.L., Cox K.E., Jeitany M., Wakimoto H., Bryll A.R., Ganem N.J., Bersani F., Pineda J.R., Suva M.L., Benes C.H. (2015). Alternative lengthening of telomeres renders cancer cells hypersensitive to ATR inhibitors. Science.

[67] Clynes D., Jelinska C., Xella B., Ayyub H., Scott C., Mitson M., Taylor S., Higgs D.R., Gibbons R.J. (2015). Suppression of the alternative lengthening of telomere pathway by the chromatin remodelling factor ATRX. Nat. Commun..

[68] Coluzzi E., Buonsante R., Leone S., Asmar A.J., Miller K.L., Cimini D., Sgura A. (2017). Transient ALT activation protects human primary cells from chromosome instability induced by low chronic oxidative stress. Sci. Rep..

[69] Russell J.S., Brady K., Burgan W.E., Cerra M.A., Oswald K.A., Camphausen K., Tofilon P.J. (2003). Gleevec-mediated inhibition of Rad51 expression and enhancement of tumor cell radiosensitivity. Cancer Res..

[70] Lee E.S., Won Y.J., Kim B.C., Park D., Bae J.H., Park S.J., Noh S.J., Kang Y.R., Choi S.H., Yoon J.H. (2016). Low-dose irradiation promotes Rad51 expression by down-regulating miR-193b-3p in hepatocytes. Sci. Rep..

[71] Bo H., Ghazizadeh M., Shimizu H., Kurihara Y., Egawa S., Moriyama Y., Tajiri T., Kawanami O. (2004). Effect of ionizing irradiation on human esophageal cancer cell lines by cDNA microarray gene expression analysis. J. Nippon Med. Sch. = Nippon Ika Daigaku Zasshi.

[72] Caslini C., Connelly J.A., Serna A., Broccoli D., Hess J.L. (2009). MLL associates with telomeres and regulates telomeric repeat-containing RNA transcription. Mol. Cell. Biol..

[73] Gottschling D.E. (1992). Telomere-proximal DNA in Saccharomyces cerevisiae is refractory to methyltransferase activity in vivo. Proc. Natl. Acad. Sci. USA.

[74] Boivin A., Dura J.M. (1998). In vivo chromatin accessibility correlates with gene silencing in Drosophila. Genetics.

[75] Murr R., Loizou J.I., Yang Y.G., Cuenin C., Li H., Wang Z.Q., Herceg Z. (2006). Histone acetylation by Trrap-Tip60 modulates loading of repair proteins and repair of DNA double-strand breaks. Nat. Cell Biol..

[76] Bao Y., Shen X. (2007). Chromatin remodeling in DNA double-strand break repair. Curr. Opin. Genet. Dev..

